# Spinal Cord and Spinal Nerve Root Involvement (Myeloradiculopathy) in Tuberculous Meningitis

**DOI:** 10.1097/MD.0000000000000404

**Published:** 2015-01-26

**Authors:** Rahul Gupta, Ravindra Kumar Garg, Amita Jain, Hardeep Singh Malhotra, Rajesh Verma, Praveen Kumar Sharma

**Affiliations:** From the Department of Neurology (RG, RKG, HSM, RV, PKS); and Department of Microbiology (AJ), King George Medical University, Lucknow, Uttar Pradesh, India.

## Abstract

Most of the information about spinal cord and nerve root involvement in tuberculous meningitis is available in the form of isolated case reports or case series. In this article, we evaluated the incidence, predictors, and prognostic impact of spinal cord and spinal nerve root involvement in tuberculous meningitis.

In this prospective study, 71 consecutive patients of newly diagnosed tuberculous meningitis were enrolled. In addition to clinical evaluation, patients were subjected to magnetic resonance imaging (MRI) of brain and spine. Patients were followed up for at least 6 months.

Out of 71 patients, 33 (46.4%) had symptoms/signs of spinal cord and spinal nerve root involvement, 22 (30.9%) of whom had symptoms/signs at enrolment. Eleven (15.4%) patients had paradoxical involvement. Paraparesis was present in 22 (31%) patients, which was of upper motor neuron type in 6 (8.4%) patients, lower motor neuron type in 10 (14%) patients, and mixed type in 6 (8.4%) patients. Quadriparesis was present in 3 (4.2%) patients. The most common finding on spinal MRI was meningeal enhancement, seen in 40 (56.3%) patients; in 22 (30.9%), enhancement was present in the lumbosacral region. Other MRI abnormalities included myelitis in 16 (22.5%), tuberculoma in 4 (5.6%), cerebrospinal fluid (CSF) loculations in 4 (5.6%), cord atrophy in 3 (4.2%), and syrinx in 2 (2.8%) patients. The significant predictor associated with myeloradiculopathy was raised CSF protein (>250 mg/dL). Myeloradiculopathy was significantly associated with poor outcome.

In conclusion, spinal cord and spinal nerve root involvement in tuberculous meningitis is common. Markedly raised CSF protein is an important predictor. Patients with myeloradiculopathy have poor outcome.

## INTRODUCTION

Outcome in patients with tuberculous meningitis has not remarkably improved in the past half century.^1^ Corticosteroids are likely to improve mortality, but do not reduce disabilities in survivors. Spinal cord and spinal nerve root involvement, together termed as myeloradiculopathy, is a major cause of disability in these patients.^2^

Tuberculous arachnoiditis is usually the cause of spinal cord and spinal nerve root involvement. Tuberculous arachnoiditis is different from other types of arachnoiditis because it frequently affects spinal cord, meninges, and nerve roots together in varying combinations.^3^ Spinal cord and spinal nerve root involvement in tuberculous meningitis presents in various forms, such as tuberculous radiculomyelitis, spinal tuberculoma, myelitis, syringomyelia, vertebral tuberculosis, and spinal tuberculous abscess, in varying frequency. Manifestations, related to the spinal cord and spinal nerve root involvement, may be present initially or appear paradoxically, following antituberculosis treatment.^4^ In tuberculous meningitis, spinal cord and spinal nerve root involvement occurs because of 3 possible pathogenic mechanisms: hematogenous spread of mycobacteria to the parenchyma and meninges of spinal cord; gravitation of tuberculous exudate to the lumbosacral region; and rarely, by direct extension from vertebral tuberculosis.^5,6^

Cameron,^7^ in 1919, had noted spinal cord and spinal nerve root involvement in tuberculous meningitis presenting as asymmetric areflexic paraparesis. This entity was initially confused with poliomyelitis, but on postmortem examination, foci of caseation among the issuing nerve roots of the lumbar enlargement were observed.^7^ Since then many reports about spinal cord and nerve root involvement in tuberculous meningitis are available, majority inform of isolated case reports or small case series.^4^

In this study, we prospectively evaluated the incidence, predictors, and prognostic impact of spinal cord and spinal nerve root involvement in patients with tuberculous meningitis.

## MATERIAL AND METHODS

This prospective study was conducted in the Department of Neurology, King George Medical University, Lucknow, Uttar Pradesh, India, a tertiary care referral facility. A written informed consent was obtained from all the patients or their relatives before inclusion in this study. Institutional ethics committee approval was taken. Patients were enrolled from November 2011 to November 2013.

### Inclusion and Exclusion Criteria

All consecutive newly diagnosed patients of tuberculous meningitis, fulfilling the consensus diagnostic criteria as described by Marais et al,^8^ were included. Accordingly, the cases were classified as definite, probable, or possible cases, depending on their total diagnostic score. Definite cases were those that showed direct evidence of acid-fast bacilli (AFB) in cerebrospinal fluid (CSF), by staining, culture, or commercial nucleic acid amplification tests. Probable tuberculous meningitis were those cases that showed a diagnostic score of ≥12 when imaging was available and a diagnostic score of ≥10 when imaging was not available. For a possible case of tuberculous meningitis, a diagnostic score of 6–11 was required when imaging was available and a score of 6–9 when imaging was not available.^9^

The patients of vertebral tuberculosis (Pott's spine) and prolapsed intervertebral disc were excluded from the study. Deeply comatose (Glasgow coma scale [GCS] score ≤10) patients were included in the study if they regained consciousness within 7 days of hospitalization. The baseline GCS score, British Medical Research Council (BMRC) staging, and modified Barthel index score were recorded for comparison at follow-up.

### Clinical Assessment

In all patients, detailed history and neurological examination were performed. The patients were asked about weakness, thinning of limbs, stiffness, abnormal movements, radicular pain, numbness, or paresthesias in extremities, band-like sensation or zone of hyperaesthesia over trunk, constipation, and any voiding (hesitancy, intermittency, straining, and retention) or storage symptoms (frequency, urgency, and urge incontinence) of micturition. Similarly, patients were examined for the presence or absence of wasting, weakness, tone, reflex, and sensory changes.

The patients were classified according to the BMRC staging system. In stage 1, patients had normal sensorium with no focal neurological deficit; in stage 2, patients had slight clouding of sensorium (GCS score 11–14), and a minor neurological deficit (such as cranial nerve palsy) or no deficit; and in stage 3, patients had severe impairment of sensorium (GCS score ≤10), convulsions, severe focal neurological deficit (hemiplegia or paraplegia), or multiple cranial nerve palsies. Baseline GCS score was recorded at the time of hospitalization and divided into 3 categories: GCS score ≤10, 11–14, and 15. Patient's baseline disability status was recorded utilizing the 10-item modified Barthel index score (maximum score – 20).^10^

### Laboratory Investigations

A battery of laboratory investigations was performed in all patients. This battery included complete hemogram, liver and renal function test, serum electrolytes, erythrocyte sedimentation rate, human immunodeficiency virus status, and chest X-ray. In all patients, CSF analysis was done for protein, total leukocyte and differential leukocyte counts, sugar with simultaneous measurement of plasma sugar, AFB stain, standard culture, and nucleic acid amplification test. India ink preparation was done to rule out cryptococcal meningitis.

### Case Definitions

Radiculopathy was defined by nerve root pain, weakness and wasting of muscles conforming to radicular distribution, sensory loss in dermatomal pattern, and asymmetrically absent deep tendon jerks. Myelopathy was defined by paraplegia or quadriplegia, presence of a sensory level below the level of the lesion, and bladder involvement.^11,12^

### Neuroimaging

In all patients, magnetic resonance imaging (MRI) of the brain and spine, with contrast, was done at the time of inclusion. Mid-sagittal T2-weighted sequence along with precontrast and postcontrast T1-weighted imaging of the whole spine were done using Signa Excite 1.5 Tesla instrument (General Electric Medical Systems, Milwaukee, WI). The scans were analyzed and reported independently by a neuroradiologist who was unaware of the clinical details of the patients.

MRI of the brain was evaluated for the presence of meningeal enhancement, basal exudates, hydrocephalus, tuberculoma, and infarct. Exudates were searched for specifically in the basal cisterns and intensely enhancing basal exudates were noted as “spider-leg appearance.” Hydrocephalus was defined by Evan index (ratio of maximal width of frontal horns to maximal width of inner skull). Hydrocephalus was labeled if the ratio was >30% or if the temporal horn was dilated by >2 mm.^13^ Tuberculoma, in both brain and spine, was identified by a predominant hypointense signal in both T1 and T2-weighted imaging and intense (solid noncaseating granuloma) or rim (caseating granuloma) enhancement.^11^

MRI of the spine was reviewed for the presence of myelitis, lumbosacral arachnoiditis, CSF loculations, tuberculoma, cord atrophy, syrinx formation, and spinal meningeal enhancement. These features were assessed by seeing an alteration in CSF signal, CSF–cord interface, signal intensity of cord in T2-weighted image, cord expansion, and nodular thickening involving the subarachnoid space. CSF and cord signal intensity alterations were decided on the basis of visual impression by comparing the same with the adjoining spinal cord. The extent of spinal involvement was assessed by the proportions of CSF signal alteration, contrast enhancement of the meninges, or clumping of nerve roots, whichever was more extensive.^12^ The presence of low signal intensity on T1-weighted imaging and high signal intensity on the corresponding region of T2-weighted imaging with a well-defined margin was taken as an evidence of syrinx formation. Myelitis was identified by hyperintense signal on T2-weighted image associated with cord edema, enlargement, and marginal enhancement on contrast.^14^ CSF loculations were delineated as extramedullary fluid loculations, having similar signal intensity as of CSF in all MRI sequences.

Lumbosacral arachnoiditis was identified by irregularity of thecal sac, nodularity and thickening of nerve roots, and their clumping.^15^ It was classified into 1 of the 3 patterns of involvement as per Delamarter classification: central conglomerations of cauda equine nerve roots; peripheral clumping of nerve roots giving empty thecal sac appearance; and soft tissue mass replacing subarachnoid space giving rise to a central opacity.^16^ MRI of the spine was repeated only in those patients who developed new symptoms suggestive of spinal cord and spinal nerve root involvement.

### Treatment

All patients were treated with antituberculosis drugs as per the recommendations of the World Health Organization for the treatment of central nervous system tuberculosis.^17^ The patients received 2 months of daily oral isoniazid (5 mg/kg of body weight; maximum 300 mg), rifampicin (10 mg/kg; maximum 600 mg), pyrazinamide (25 mg/kg; maximum 2 g/d), and intramuscular streptomycin (20 mg/kg; maximum 1 g/d) followed by 7 months of isoniazid and rifampicin at the same daily dose. All patients received 8 weeks of dexamethasone, which included 4 weeks of intravenous dexamethasone starting at a dose of 0.4 mg/kg for 1 week and gradually tapering it off by 0.1 mg/kg/wk over the next 3 weeks, followed by 4 weeks of oral steroids starting at a dose of 4 mg/d for 1 week and gradually tapering it off by 1 mg/wk over the next 3 weeks.^18^ The other drugs used were intravenous mannitol, acetazolamide, and diuretics for raised intracranial pressure or hydrocephalus; antiepileptic drugs for seizures; and pyridoxine for prophylaxis against isoniazid-induced peripheral neuropathy in high-risk patients.

### Follow-Up and Assessment of Outcome

All patients were reassessed at 1, 3, and 6 months for improvement or deterioration of their clinical status as assessed by the modified Barthel index score. For statistical analysis, the outcome was defined as good if modified Barthel index score was ≥12, and poor if either the patient died or modified Barthel index score was <12.^19^

### Statistical Analysis

The statistical analysis was performed using the Statistical Package for Social Sciences, Version 16.0 for Windows (SPSS, Chicago, IL) and Microsoft Excel. Predictors were identified using univariate and multivariate analyses. Univariate analysis was performed by χ^2^ test for nonparametric data and Student “*t*” test for independent variables for parametric data; relative risks with 95% confidence interval were ascertained. For multivariate analysis, binary logistic regression was performed to see the significance of results. Kaplan–Meier analysis was performed to estimate the event-free survival for the outcome with or without tuberculous myeloradiculopathy using the log rank test. Statistical significance was defined at a *P* value of <0.05 and wherever analysis was done it was 2 tailed.

## RESULTS

**Figure 1:**
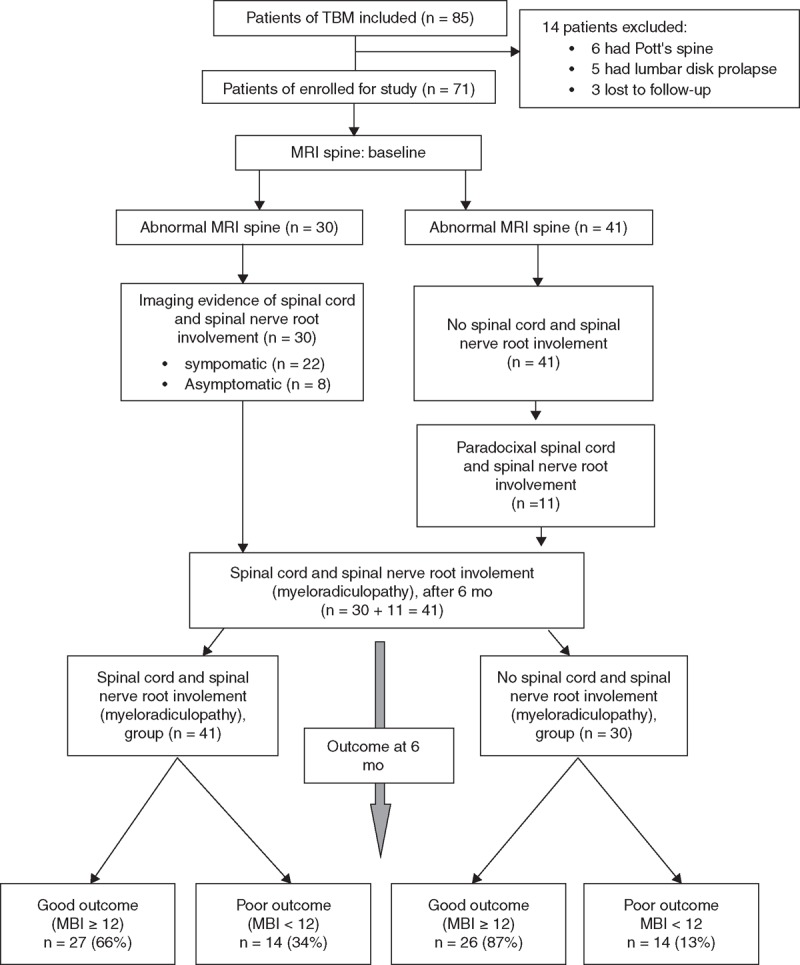
Flow chart of the study (MBI= modified Barthel index; TBM= tuberculous meningitis).

Eighty-five patients, who fulfilled the diagnostic criteria for tuberculous meningitis, were considered for enrolment in the study. Fourteen patients were excluded. Finally, 71 patients were analyzed as per the study protocol (Figure 1). Baseline clinical, neuroimaging, diagnostic category, staging of tuberculous meningitis, and baseline disability status of enroled patients have been described in Table 1. Two patients were positive for human immunodeficiency virus.

**Table 1:**
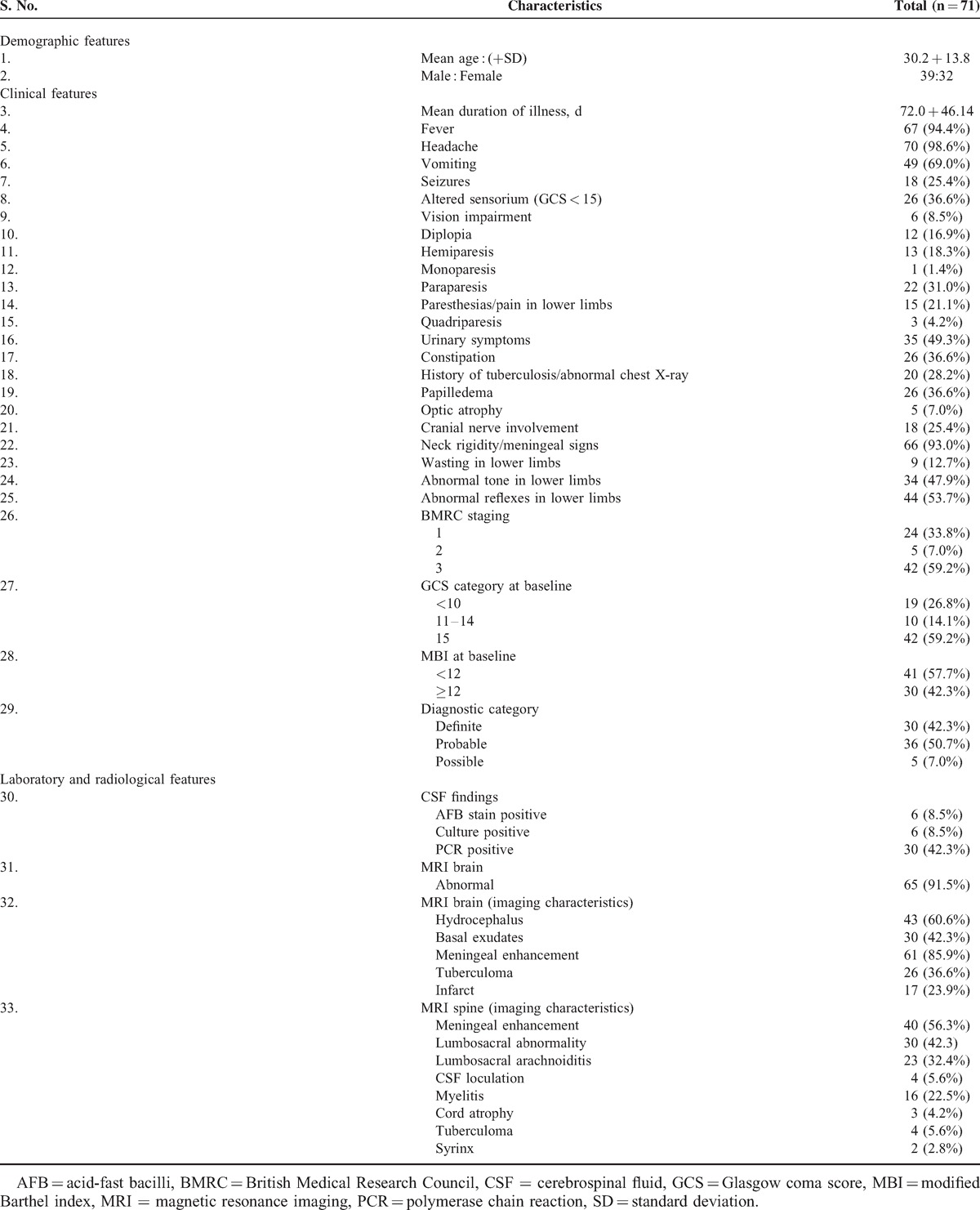
Baseline Epidemiological, Clinical, and Neuroimaging Characteristics of Patients With Tuberculous Meningitis (n = 71)

### Spinal Cord and Spinal Nerve Root Involvement

Out of 71 patients, 33 (46.4%) patients had symptoms/signs suggestive of spinal cord and spinal nerve root involvement. Twenty-two (30.9%) patients had symptoms at enrolment. Eleven (15.4%) patients paradoxically developed symptoms during follow-up. Common presenting symptoms were weakness, pain, and paresthesias in lower limbs, urinary complaints, and constipation. The common clinical signs on neurological examination were reflex changes, tone changes, decreased power on Medical Research Council scale, extensor plantar response, and sensory loss. Paraparesis was present in 22 (31%) patients; it was of upper motor neuron (UMN) type in 6 (8.4%), lower motor neuron type in 10 (14%), and mixed type in 6 (8.4%) patients. Quadriparesis was present in 3 (4.2%) patients; it was of UMN type in 2 patients and mixed type in 1 patient. Monoparesis was present in a single patient (computed tomography showed infarct in the brain) (Table 2 ).

**Table 2:**
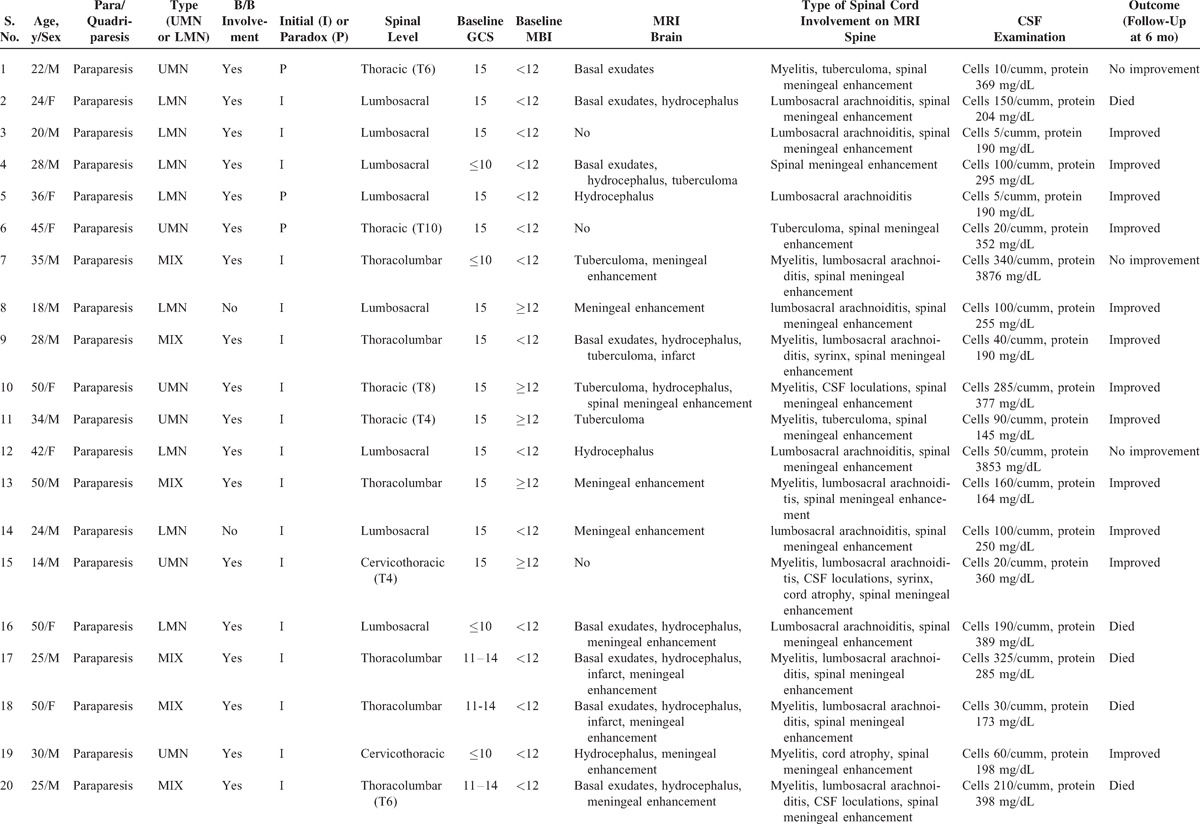
Clinicoradiological and Outcome Details of Patients With Either Quadriparesis or Paraparesis

**Table 2 (Continued):**
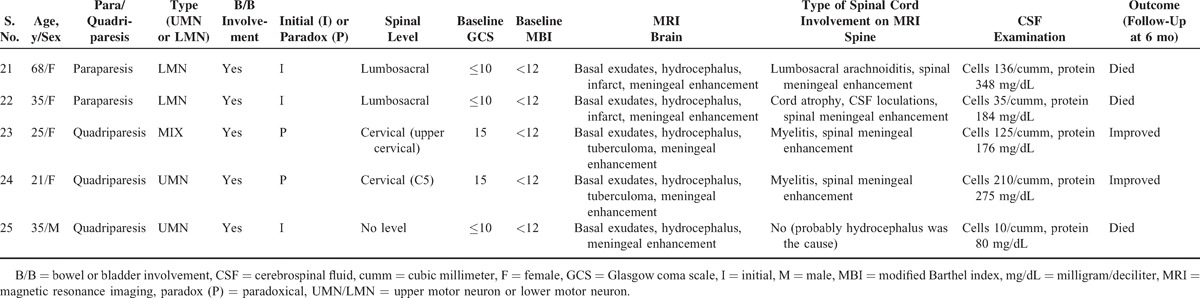
Clinicoradiological and Outcome Details of Patients With Either Quadriparesis or Paraparesis

### MRI Spine Findings

Forty-one (57.7%) patients had spinal cord and/or spinal nerve root changes on MRI that were present in the form of myelitis, lumbosacral arachnoiditis, tuberculoma, syrinx, cord atrophy, CSF loculations, and spinal meningeal enhancement, either alone or in combination. The most common site of involvement was lumbosacral region (23, 32.4%), followed by thoracolumbar (7, 9.9%), thoracic (6, 8.5%), cervical (3, 4.2%), and cervicothoracic and cervicothoracolumbar regions in 1 patient each. Both the human immunodeficiency virus-positive patients had dorsal cord involvement.

**Figure 2:**
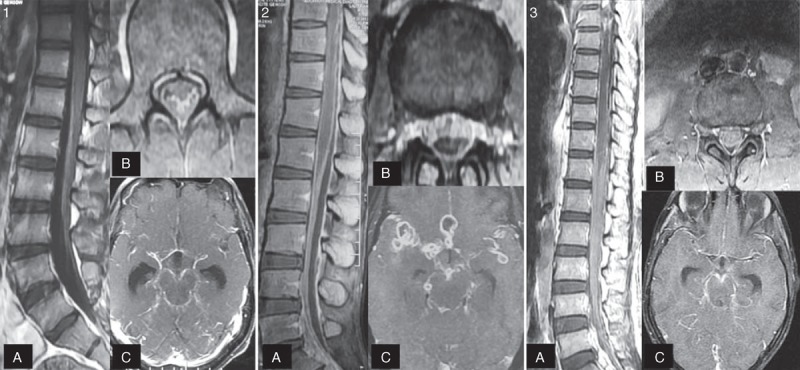
Gadolinium-enhanced T1-weighted MRI of the brain (axial sections) and spine (sagittal and axial sections, fat suppressed) depict different pattern of involvement in 3 patients (1ABC, 2ABC, and 3ABC) with tuberculous affliction of the brain and spine. Figure 1A and 1B show lumbosacral arachnoiditis with conglomeration of nerve roots in center of thecal sac (Delamarter's group 1) at L3 vertebral level, and Figure 1C shows meningeal enhancement at the level of interpeduncular cistern. Figure 2A and 2B depict peripheral clumping of nerve roots at L3-L4 vertebral level giving rise to empty thecal sac appearance (Delamarter's group 2). Figure 2C demonstrates meningeal enhancement and tuberculomas at the level of interpeduncular cistern. Figure 3A and 3B show complete opacification of the subarachnoid space (Delamarter's group 3) at L3 vertebral level. Figure 3C reveals meningeal enhancement at the level of interpeduncular cistern. MRI = magnetic resonance imaging.

**Figure 3:**
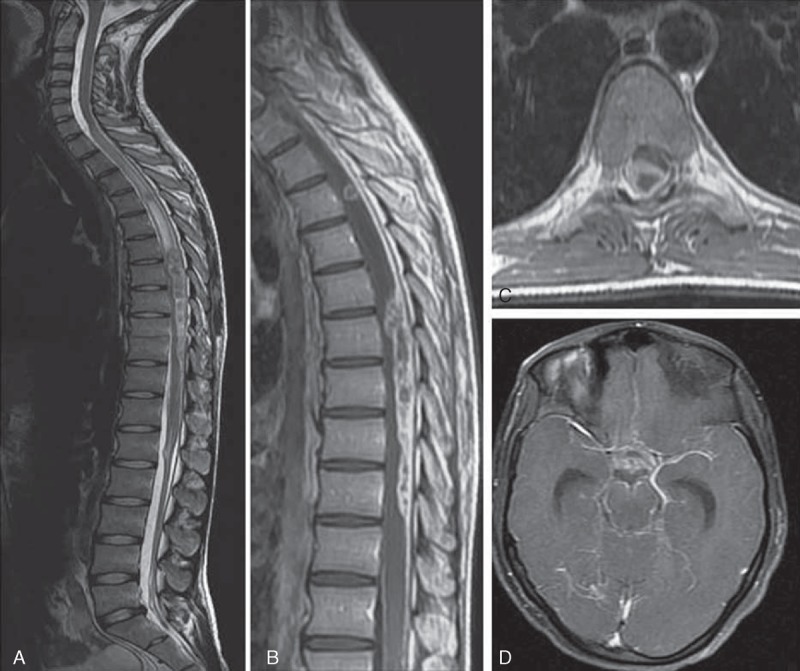
MRI spine of a 22-year-old man with tuberculous meningitis, the patient presented with paraparesis, of 4 weeks duration. T2-weighted image shows long segment myelitis extending from D3 to D6 thoracic level (A); a rounded intramedullary hypointensity at D4 level showing ring enhancement in postcontrast fat suppression imaging (B) and intradural–extramedullary collection extending from D6 to D10 level (A–C). Also, there is meningeal enhancement surrounding the conus medullaris (B). MRI brain of the same patient revealed basal meningeal enhancement and hydrocephalus (D). MRI = magnetic resonance imaging.

**Figure 4:**
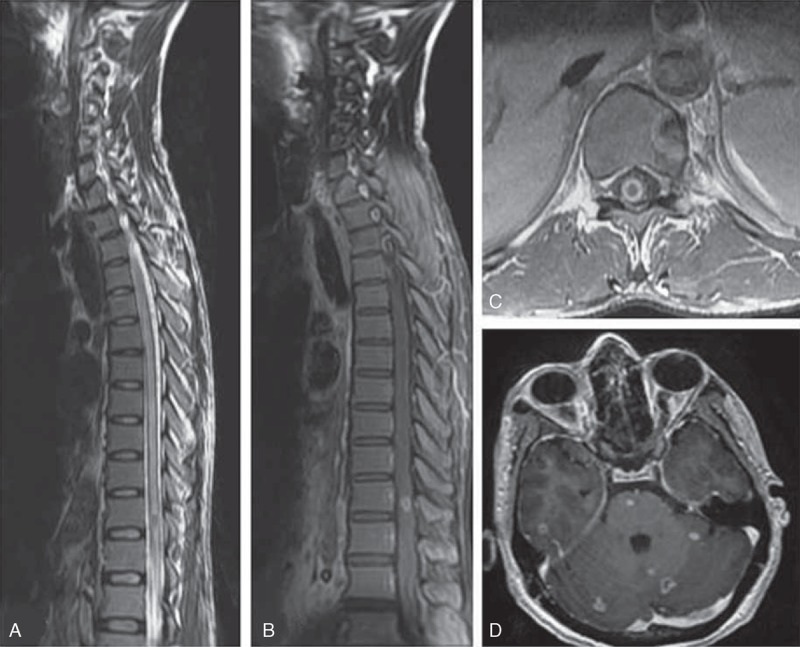
MRI spine of a 55-year-old man with tuberculous meningitis, who presented with a 3 weeks history of paraparesis, MRI showed long segment myelitis (A) and intramedullary tuberculoma at D10 level (B and C). MRI brain of the patient showed multiple tuberculoma involving cerebrum, cerebellum, and brainstem (D). MRI = magnetic resonance imaging.

The most common finding on MRI of the spine was meningeal enhancement, seen in 40 (56.3%) patients. Spinal meningeal enhancement was present in lumbosacral region in 22 (30.9%) patients, thoracolumbar region in 7 (9.8%), thoracic in 6 (8.4%), and cervical in 3 (4.2%); involvement of cervicothoracic and cervicothoracolumbar region was observed in 1 (1.4%) patient each (Figure 2). In addition to this, MRI abnormalities included myelitis in 16 (22.5%), tuberculoma in 4 (5.6%), CSF loculations in 4 (5.6%), cord atrophy in 3 (4.2%), and syrinx in 2 (2.8%) patients. Myelitis was in dorsal cord in 12 (16.9%), cervical in 2 (2.8%), and cervicodorsal in 2 (2.8%) patients. All 4 tuberculoma were noted in the dorsal cord; in 2 (2.8%) patients, it was extramedullary and in another 2 (2.8%), it was intramedullary. CSF loculations were seen in the cervical region in 2 (2.8%) patients and in the thoracic region in another 2 (2.8%) patients. Syrinx was observed in the cervicothoracic region in 2 (2.8%) patients (Figures 3 and 4).

In lumbosacral arachnoiditis, 4 patterns of involvement were recorded according to Delamarter classification. In 4 (5.6%) patients, conglomeration of radicles was seen in the center of thecal sac; in 9 (12.7%), clumping of nerve roots was seen at peripheral part of meninges leading into empty thecal sac appearance; in 8 (11.3%), there was soft tissue mass replacing subarachnoid space and in 2 (2.8%), a mixed pattern was seen at different levels of lumbosacral spine (Figure 2).

Thirty-three patients were either symptomatic or had clinical signs that suggested myeloradicular involvement. Eight patients (11.3%) had asymptomatic spinal meningeal enhancement: 6 (8.4%) at lumbosacral, 1 (1.4%) at thoracic, and another (1.4%) at thoracolumbar region.

### Predictors of Spinal Cord and Nerve Root Involvement

On univariate analysis, significant predictors associated with spinal cord and/or spinal nerve root involvement were raised CSF protein of >250 mg/dL and baseline modified Barthel index <12 (Tables 3 and 4). On multivariate analysis, none of the factors was found to be significant.

**Table 3:**
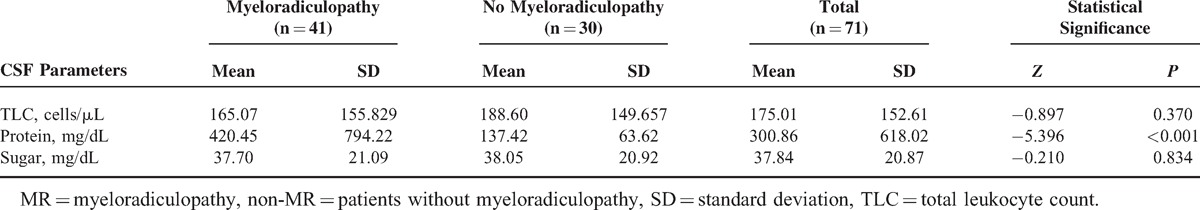
Mean Baseline CSF Parameters of Patients With and Without Myeloradiculopathy

**Table 4:**
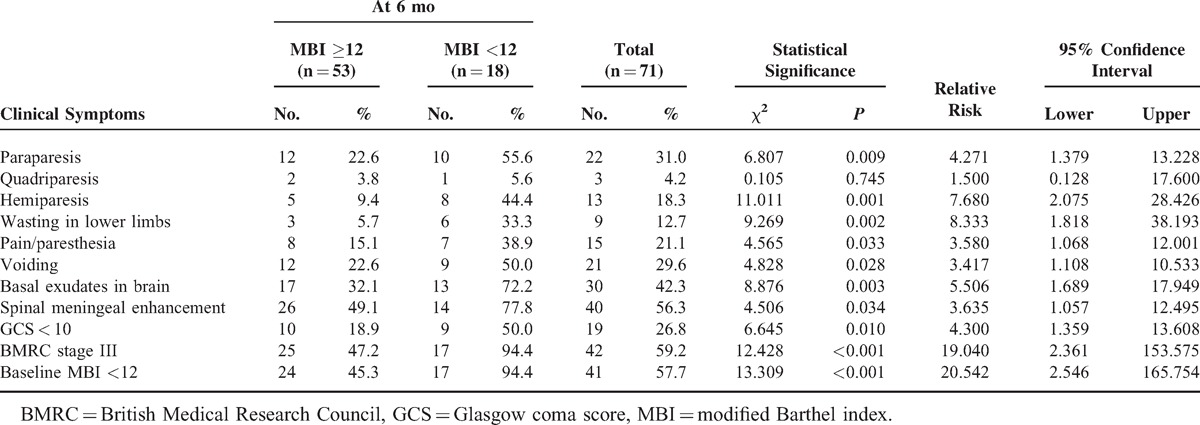
Clinical and Neuroimaging Predictors of Poor Outcome

### Follow-Up at 6 Months

At 6 months, 53 (74.6%) patients had good outcome (modified Barthel index ≥12). Eighteen (25.3%) patients had poor outcome, out of which, 13 (18.3%) patients died and 5 (7.0%) had severe disability (modified Barthel index <12). Out of 41 patients in spinal cord and/or spinal nerve root involvement group, 27 (65.9%) had good outcome whereas 14 (34.1%) had poor (modified Barthel index <12 or died). Out of 33 patients who were symptomatic for myeloradiculopathy, 12 had poor outcome whereas among 8 patients, who had asymptomatic spinal meningeal enhancement, 2 had poor outcome. In patients without myeloradiculopathy, 26 (86.7%) had good outcome whereas 4 (13.3%) had poor (Figure 1). This was statistically significant (*P* = 0.046) on univariate analysis.

### Clinical and Neuroimaging Predictors of Poor Outcome

**Figure 5:**
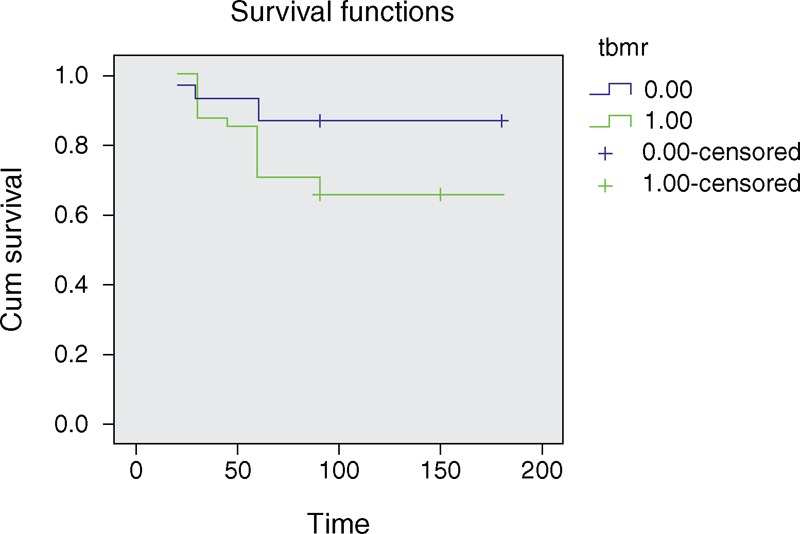
Survival functions in patients of tuberculous meningitis with or without myeloradiculopathy.

Kaplan–Meier analysis showed that myeloradiculopathy was significantly associated with poor outcome, but the difference was not significant on logistic regression (Figure 5). On univariate analysis, wasting in lower limbs, paraparesis, hemiparesis, paresthesias in lower limbs, voiding symptoms on urination, basal exudates on MRI, spinal meningeal enhancement on MRI, low baseline GCS score (<15), low baseline modified Barthel index score (<12), and stage 3 of BMRC staging were significantly associated with poor outcome. On multivariate analysis of these clinical and neuroimaging parameters, only hemiparesis was significantly associated with poor outcome (Table 4).

## DISCUSSION

We observed that a large number of patients with tuberculous meningitis either have or paradoxically develop clinical manifestations of spinal cord and spinal nerve root involvement. In addition to this, neuroimaging findings (arachnoiditis and myelitis) were also noted in more than half of these patients. Our findings are important because, spinal cord and spinal nerve root involvement significantly contribute to the disability of patients, which, possibly, is often overlooked. We also noted that any part of the spinal cord may be involved but the lumbosacral region is most frequently affected, where the presence of exudates result in clumping of nerve roots.

Approximately, 10% of cases with tuberculous meningitis are supposed to have some form of spinal tuberculosis.^20^ We in our study observed that quite a large proportion of patients of tuberculous meningitis have spinal and/or radicular syndrome. Brooks et al,^21^ in 1954, reported that out of 80 patients with tuberculous meningitis, 15 developed spinal block and 10 developed transverse myelitis. Spinal block, in this series, was diagnosed when a Froin syndrome (CSF protein >500 mg/dL) and a negative Queckenstedt response were present.^21^ In an another report, paraplegia occurred in 8 of 17 patients with central nervous system tuberculosis.^22^ Paraplegia due to spinal cord tuberculosis is uncommon in developed countries. In New Zealand, among 104 patients with definite or probable tuberculous meningitis, myeloradiculopathy causing sphincter dysfunction, and lower limb weakness and sensory loss occurred in 3 patients. The CSF protein concentration was >5 g/L in 2 of these patients. Myeloradiculopathy was a late complication in 2 patients, but in the other patient, paraplegia and urinary retention developed 10 days after admission. One patient had presented 3 years after he was treated for tuberculous meningitis with a progressive neurological deficit caused by syringomyelia.^23^ We feel that spinal cord and spinal nerve root involvement is an integral part of tuberculous meningitis manifestations and can occur at a variable frequency. The longer mean duration of illness (72 days in our study) before the diagnosis, and subsequent delay in the initiation of chemotherapy, might be the factors contributing to the higher proportion of myeloradicular complications in this part of the world as compared with the developed world. Poor nutritional status, lack of a uniform policy in administrating antituberculosis drugs (dose, regime, and duration) at various levels of health care professionals, and a differential response to drugs may also be important.

In addition to lumbosacral arachnoiditis, a variety of other spinal syndromes have been observed in patients with tuberculous meningitis. These include myelitis with cord edema, intramedullary or extramedullary tuberculoma, and, infrequently, formation of a syrinx. Extramedullary spinal granulomas without bony involvement and intramedullary spinal tuberculoma are often paradoxical manifestations of tuberculous meningitis. Intraspinal cord abscess is a rare complication of tuberculous meningitis. Spinal tuberculomas may cause confusion with spinal cord neoplasm. The cause of acute syringomyelia in tuberculous meningitis has been ascribed to thrombosis and endarteritis of the spinal cord vessels leading to softening of the spinal cord and subsequent myelomalacia.^4^ In our patients, the most common MRI spine finding was spinal meningeal enhancement, lumbosacral arachnoiditis, and thoracic myelitis. The less-common findings were CSF loculations, tuberculoma, and syrinx. The damage to the cord, mainly the white matter, occurs through edema and ischemia, rather than frank infarction, the larger arteries being rarely involved.^24–26^

Before MRI era, postmortem evaluation of these patients, frequently, noted involvement of lumbosacral meninges and cauda equina. Pathologically, it has been observed that the subarachnoid space between the spinal dura mater and the leptomeninges may be filled with thick gelatinous exudate and this encases the spinal cord and emerging nerve roots. The cord and nerve roots are inflamed and are edematous. In due course, thick exudates get organized and fibrin-coated nerve roots stick to each other as well as to the meninges. Small tuberculous granulomas were noted on the meninges as well as in the parenchyma of the spinal cord. Vasculitis of the spinal arteries may lead to spinal cord ischemia.^5,6,27^

On univariate analysis, markedly increased CSF protein was significantly associated with spinal cord and spinal nerve root involvement. In patients of tuberculous meningitis with spinal cord and nerve root involvement, a high level of CSF protein possibly results from the spinal block produced by thick tenacious exudate.^28^ Spinal arachnoiditis may even be asymptomatic. In a study that included 16 patients with tuberculous meningitis of <1 month duration, spinal MRI revealed evidence of asymptomatic spinal arachnoiditis in 3 patients. High CSF protein was a risk factor for development of spinal arachnoiditis.^29^ Tuberculous spinal cord involvement may also develop paradoxically, while patient is being treated with antituberculous drugs. Paradoxical response is defined as appearance of new tuberculoma or expansion of preexisting tuberculoma while the patient is receiving adequate antituberculous therapy is known as paradoxical response. Usually, paradoxical response and associated clinical deterioration occur several weeks after starting antituberculous therapy. Paradoxical response is thought to represent a delayed-type hypersensitivity reaction to the massive release of mycobacterial proteins into the core of tuberculoma and subarachnoid space leading to intense inflammation and expansion of tuberculoma.^4^

In our study, spinal cord and spinal nerve root involvement was associated with poor prognosis. However, the outcome of tuberculous meningitis in our study was also found to be associated with many other factors such as low baseline GCS score, baseline modified Barthel index of <12 and stage 3 of tuberculous meningitis. These factors may actually indicate either an advanced or an accelerated form of tuberculous affliction.

Our study had certain limitations. Being a single-center study, the extrapolation of results to a larger population shall remain limited. Referral bias, and subsequent higher incidence/prevalence reporting of complications, cannot be ruled out at a tertiary care referral facility, as ours. Inclusion of more human immunodeficiency virus-positive patients could have provided a comprehensive comparative substrate in the study, both clinically as well as radiologically.

In conclusion, spinal cord and spinal nerve root involvement in tuberculous meningitis are common. Significantly raised CSF protein is an important predictor of spinal cord and spinal nerve root involvement. Patients with myeloradiculopathy have poor outcome.
